# Editorial: Inflammatory biomarkers in type 1 diabetes

**DOI:** 10.3389/fendo.2026.1874373

**Published:** 2026-05-22

**Authors:** Joanna Filipowska, Antonio Rossi

**Affiliations:** 1 Department of Translational Research and Cellular Therapeutics, Arthur Riggs Diabetes and Metabolism Research Institute, City of Hope, Duarte, CA, United States; 2 I.R.C.C.S. Ospedale Galeazzi - Sant'Ambrogio, Division of Internal Medicine, Endocrinology and Metabolic Diseases, Milan, Italy; 3 Department of Biomedical and Clinical Sciences, University of Milan, Milan, Italy

**Keywords:** biomarkers, inflammation, miRNA, precision care, type 1 diabetes

Type 1 diabetes (T1D) is increasingly recognized as a dynamic and progressive autoimmune disease rather than a condition that begins only at clinical diagnosis (1–3).

Despite recent advances in transcriptomic and proteomic analyses, collectively with other approaches referred as omics, the biomarker landscape in T1D remains largely dominated by autoantibody detection and metabolic indicators, such as plasma glucose levels, HbA1c, and C-peptide (4, 5). While these traditional markers are essential for disease staging and prediction, they provide limited insight into the underlying molecular mechanisms driving disease progression and heterogeneous clinical manifestations (6, 7). Increasing evidence indicates that inflammatory signaling and intrinsic β-cell stress responses play central roles in both disease initiation and progression (8–10). Consequently, there is growing interest in developing new methods (based on the omics approaches) to identify sensitive biomarkers reflecting a dynamic interplay between immune responses and pancreatic β-cells before overt β-cell destruction begins (11).

This Research Topic brings together contributions exploring emerging molecular indicators that may bridge mechanistic understanding and expand diagnostic application in T1D. From circulating microRNAs to high-resolution single-cell transcriptomics, the studies included indicate how multilayered biological signals can refine disease prediction, elucidate pathophysiological mechanisms, and shape new directions for therapy and precision medicine in T1D.

## Expanding the Biomarker Landscape

Among emerging biomarkers in T1D, microRNAs (miRNAs) have attracted considerable attention due to their regulatory roles in gene expression and their stability in biological fluids (12–14). These small non-coding RNAs influence a wide range of biological processes, including immune activation, metabolic regulation, and cellular stress responses (12–15).

In this Research Topic, Zhang et al. summarize the significance of miRNAs in the development and progression of T1D. The authors highlight how miRNA networks regulate pathways involved in β-cell apoptosis, immune signaling, and inflammatory responses. Several miRNAs discussed in the paper, including miR-21, the miR-29 family, miR-375, miR-7, and miR-146a, function both as circulating biomarkers and potential therapeutic targets. Their altered expression reflects β-cell stress, immune activation, and disease progression, while modulation of these miRNAs may help reduce inflammation, protect β-cell or promote their regeneration. As miRNAs can be detected in circulation, they represent promising minimally invasive biomarkers capable of reflecting early disease processes. Alterations in circulating miRNA profiles may precede clinical disease onset by several years, suggesting their potential as early indicators of both immune activation and intrinsic β-cell dysfunction. However, several limitations hinder the clinical application of miRNAs in T1D. miRNA regulatory networks are highly complex, detection requires strict and rapid sample processing, and miRNA-based therapies may cause off-target effects and toxicity. Thus, there is a need for further validation and well-designed clinical studies before diagnostic implementation.

Beyond disease initiation, systemic presence of selected miRNAs may also contribute to the development of long-term complications associated with T1D. Cardiovascular disease remains a leading cause of morbidity and mortality in individuals with diabetes, and vascular alterations often begin early in life. Even during childhood, patients with T1D may develop endothelial dysfunction, arterial stiffness, and increased carotid intima-media thickness (16). Peczynska et al. focus on the role of circulating and exosomal miRNAs in cardiovascular risk among children and adolescents with T1D. In subjects with T1D, dysregulated miRNAs associated with cardiovascular disease include miR-21, miR-29, miR-34a, miR-126, miR-146a, miR-155, miR-222-3p, and miR-92a, which orchestrate endothelial dysfunction, inflammation, and atherosclerosis. Exosome or microvesicle-associated miRNAs implicated in cardiovascular risk include miR-21, miR-122, miR-155, miR-3129-5p, miR-20b, miR-9-5p, miR-320d, miR-301a-5p, and miR-155-5p. The authors conclude that while miRNAs are promising biomarkers, their clinical implementation requires improved assay standardization, better validation across independent cohorts, and the development of reliable miRNA panels rather than single markers to increase diagnostic specificity and reproducibility.

Dikranian et al. present a systematic review analyzing 39 studies encompassing more than 9, 000 pediatric patients to identify predictors of partial remission. This phase represents a critical therapeutic window during which disease-modifying interventions may have the greatest potential to preserve β-cell function. Improved characterization of partial remission may ultimately facilitate the development of targeted therapeutic strategies aimed at prolonging β-cell function.

The authors describe several clinical and biological markers of partial remission in pediatric T1D, including C-peptide, IDAA1c, CGM-derived metrics, immune signatures, hormones, proteomics, and microRNAs, as potential biomarkers to monitor residual β-cell function and disease trajectory. However, they underscore significant drawbacks, including heterogeneity in measurement methods, variability in cut-off definitions, influence of confounding factors (age, insulin sensitivity, treatment practices), and inconsistent correlations with clinical outcomes, which limit their reliability and comparability across studies.

Genetic susceptibility remains a cornerstone of T1D risk. While human leukocyte antigen (HLA) genes account for a substantial portion of genetic predisposition, non-HLA immune regulatory pathways are increasingly recognized as contributors to disease heterogeneity and autoimmune phenotypes.

Semzezem et al. investigated polymorphisms in the cytokine receptors IL17RA and IL21R in a cohort exceeding 1, 200 individuals (639 subjects with T1D and 653 healthy controls). IL-17RA and IL-21R are cytokine receptors involved in the Th17 inflammatory pathway, where IL-17RA mediates IL-17-driven inflammatory signaling and IL-21R regulates T- and B-cell activation, antibody production, and immune modulation. In the study, the IL17RA rs2241049 G and IL21R rs7199138 C alleles were associated with increased susceptibility to Type 1 diabetes, whereas IL17RA rs879577 AA and rs5748863 G variants showed protective associations or reduced autoantibody frequency. Clinical relevance will require validation in larger and ethnically diverse cohorts, independent replication, and functional studies confirming their effects on receptor signaling and disease mechanisms. The review by Abdullatypov et al. highlights the transformative impact of single-cell RNA sequencing (scRNA-seq) on T1D research, revealing substantial cellular heterogeneity within pancreatic islets and immune compartments. The authors discuss that scRNA-seq has revealed cellular and transcriptional heterogeneity in Type 1 diabetes, identifying disease-associated immune cell subsets and candidate biomarkers such as IL32 in T cells, TCL1A in naïve B cells, and ACTG1, REL, and TRIB1 in monocytes, as well as markers of β-cell dysfunction. However, clinical translation remains limited due to technical constraints, including transcript loss during library preparation, dependence on high-quality samples, and limited access to human pancreatic tissue. The authors therefore emphasize the need to integrate scRNA-seq with complementary transcriptomic approaches and validate biomarkers in accessible tissues such as blood. Together, these findings support the view that T1D is a complex, multicellular disease driven by interactions between genetically predisposed immune pathways and stressed β-cells long before clinical diagnosis.

## Toward Integrated Biomarker Frameworks

Type 1 diabetes is a dynamic disease driven by interactions among genetic susceptibility, immune dysregulation, and intrinsic β-cell stress.

Collectively, the contributions in this Research Topic emphasize the need to move beyond individual biomarkers toward integrated molecular frameworks that capture the complexity of T1D pathogenesis. Emerging indicators, including miRNA networks, cytokine signaling pathways, cell-specific transcriptional signatures, and genetic variants, provide complementary insights into different aspects of disease biology. Translating these findings into clinical practice will require standardized biomarker assays, validation in large and diverse patient cohorts, and effective integration into clinical workflows. Furthermore, the increasing availability of multi-omics datasets requires advanced analytical approaches capable of integrating complex molecular information.

Future research should therefore prioritize multi-marker models combining genetic, immunological, and metabolic indicators. Such approaches may help incorporate these biomarkers into clinical practice to predict disease trajectory, more accurately reflect disease heterogeneity and support the development of precision medicine strategies tailored to individual patients.
